# Hematopoietic–Mesenchymal Signals Regulate the Properties of Mesenchymal Stem Cells

**DOI:** 10.3390/ijms23158238

**Published:** 2022-07-26

**Authors:** Sanshiro Kanazawa, Hiroyuki Okada, Dan Riu, Yo Mabuchi, Chihiro Akazawa, Junichi Iwata, Kazuto Hoshi, Atsuhiko Hikita

**Affiliations:** 1 Department of Oral and Maxillofacial Surgery, Graduate School of Medicine, The University of Tokyo, 7-3-1 Hongo, Bunkyo-ku, Tokyo 113-0033, Japan; sanshirokanazawa@gmail.com; 2 Center for Disease Biology and Integrative Medicine, Graduate School of Medicine, The University of Tokyo, 7-3-1 Hongo, Bunkyo-ku, Tokyo 113-8655, Japan; hokadatky@gmail.com; 3 Department of Tissue Engineering, The University of Tokyo Hospital, 7-3-1 Hongo, Bunkyo-ku, Tokyo 113-0033, Japan; danriu2000@yahoo.co.jp (D.R.); ahikita-tky@g.ecc.u-tokyo.ac.jp (A.H.); 4 Department of Biochemistry and Biophysics, Graduate School of Medical and Dental Sciences, Tokyo Medical and Dental University, 1-5-45 Yushima, Bunkyo-ku, Tokyo 113-8510, Japan; yomabuchi1@gmail.com; 5 Intractable Disease Research Centre, Juntendo University School of Medicine, Hongo 2-1-1, Bunkyo-ku, Tokyo 113-8431, Japan; c.akazawa.gt@juntendo.ac.jp; 6 Department of Diagnostic & Biomedical Sciences, The University of Texas Health Science Center at Houston, 7000 Fannin St, Houston, TX 77030, USA; coperpot@hotmail.co.jp

**Keywords:** mesenchymal stem cells, hematopoietic stem cells, hematopoietic–mesenchymal cell interaction, hematopoietic–mesenchymal signaling

## Abstract

It is well known that the properties of hematopoietic stem/progenitor cells (HSCs), such as their self-renewal ability and multipotency, are maintained through interactions with mesenchymal stem/stromal cells (MSCs). MSCs are rare cells that are present in the bone marrow and are useful for clinical applications due to their functional ability. To obtain the necessary number of cells, MSCs must be cultured to expand, but this causes a remarkable decrease in stem cell properties, such as multipotency and proliferation ability. In this study, we show that the c-Mpl signal, which is related to the maintenance of hematopoietic stem cells, has an important effect on the proliferation and differentiation ability of MSCs. Utilizing a co-culture system comprising MSCs and HSCs, it is suggested that signaling from hematopoietic cells to MSCs supports cell proliferation. Interestingly, the enhanced proliferation ability of the HSCs was decreased in c-Mpl knock-out HSCs (c-Mpl-KO). In addition, the MSCs co-cultured with c-Mpl-KO HSCs had reduced MSC marker expression (PDGFRa and Sca-1) compared to the MSCs co-cultured with c-Mpl-wild-type HSCs. These results suggest that a hematopoietic–mesenchymal signal exists, and that the state of the HSCs is important for the stability of MSC properties.

## 1. Introduction

Bone marrow mesenchymal stem/stromal cells (MSCs) are present in the bone marrow and differentiate into several types of cells when tissue damage occurs, such as osteoblasts, chondrocytes, adipocytes, and myocytes. Owing to their multipotency, MSCs have already been clinically applied as a cell source in regenerative medicine [1]. Previous studies have reported several combinations of cell surface markers for MSCs; for example, CD90 and CD 271 can be used as markers to select cells from the bone marrow and have shown a high level of performance [2,3]. Cultured bone marrow cells expressing CD90, CD105, and CD73 are also known to have high potential for regenerative medicine [4,5]. However, MSCs have a very low probability of existence in the bone marrow (approximately one per two million), and even if MSCs can be separated using these markers, for clinical application immediately after isolation, a large amount of bone marrow is required to obtain sufficient numbers. To obtain the necessary number of cells, MSCs must be cultured to expand, but this causes a remarkable decrease in stem cell properties (stemness), such as multipotency and proliferation ability [6]. Currently, research aimed at elucidating the molecular mechanisms of MSC self-renewal and stemness maintenance is progressing [7,8]. However, it remains challenging to establish a method for culturing MSCs while also maintaining stemness. If such a method can be established, the use of MSCs in clinical applications will dramatically expand.

The mesenchymal cells in the bone marrow provide a microenvironment called a niche, in which the stemness of hematopoietic stem/progenitor cells (HSCs) is maintained by various factors, such as C-X-C motif chemokine ligand 12 (CXCL12) and hyaluronic acid [9,10,11]. These mesenchymal cells are presumed to correspond to nestin-positive cells, N-cadherin-positive cells, CD31-positive cells, and CXCL12-expressing reticular cells [12,13,14,15]. It is well known that thrombopoietin (TPO) is essential for megakaryopoiesis [16] and contributes to the maintenance and expansion of HSCs [17,18,19]. Mice deficient in TPO, or its receptor (c-Mpl), not only show impaired megakaryopoiesis but also reduced HSC number and function [20,21]. Moreover, recent reports have indicated that TPO is required for the maintenance of HSCs in a quiescent state within the bone marrow [22,23]. 

Cell–cell interactions play pivotal roles in the various developmental phases and the maintenance of tissue homeostasis. Such interactions are often observed among cells from different germ layers during ontogeny and organogenesis, such as the interaction of heart fields and the splanchnic endoderm and the conjugative generation of epithelial enamel and mesenchymal dentin in tooth germ [24,25]. A recent report indicated that a stemness signal from the HSCs to MSCs is also present as a counterpart of the stemness signals of the HSCs of mesenchymal lineage [26,27,28]. Therefore, we hypothesized that TPO/c-Mpl signals are also important for the stemness signals from the HSCs to mesenchymal cells. In this study, we utilized c-Mpl-deficient mice to examine the impact of c-Mpl deficiency in both HSCs and MSCs on their proliferation ability and maintenance of stemness.

## 2. Results

### 2.1. Proliferation and Chondrogenic Potential of Mesenchymal Cells Increase during Co-Culture with Hematopoietic Cells

**Figure 1:**
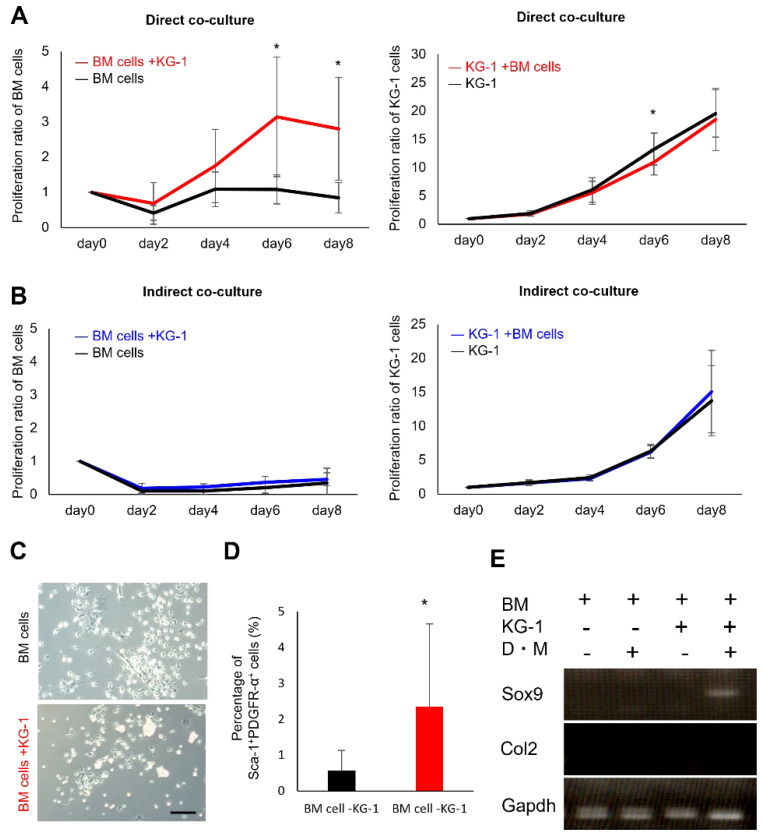
Mesenchymal–hematopoietic cell interaction promotes BM cell proliferation. (**A**) Cell proliferation rate of BM cells (left) and KG-1 cells (right). Direct co-culture of BM cells and KG-1 cells (cells were added directly). The number of cultured cells was determined (*n* = 5). * *p* < 0.05. (**B**) Cell proliferation curve by indirect culture of BM cells (left) and KG-1 cells (right). Indirect co-culture of BM cells and KG-1 cells using culture inserts. (**C**) Microscopic views of BM cells cultured alone (left) or co-cultured with KG-1 cells directly (right). Scale bar = 100 µm. (**D**) Flow cytometry analysis of cell surface markers in BM cells cultured alone or co-cultured directly with KG-1 cells. The graph shows the averages of the percentages of Sca-1 and PDGFR-α double-positive cells (*n* = 5). * *p* < 0.05. (**E**) RT-PCR analysis of chondrogenic differentiation marker (Sox9 and Col2) gene expression in BM cells following a 2-week treatment with chondrogenic differentiation media (indicated as D.M) or control media after co-culture with KG-1 cells (indicated as KG-1) or monoculture.

To verify the existence of hematopoietic–mesenchymal cell interaction, we co-cultured bone marrow-derived mesenchymal cells (BM cells) from EGFP-expressing mice with human acute leukemia cells (KG-1 cell line). The reason for using KG-1 in this study was to screen for the presence of interactions between mesenchymal cells and hematopoietic cells. In addition, cell lines would be highly useful for an initial study, as these cells offer more versatility than primary cells. We counted the number of cells in the co-culture over time and found that the proliferation of BM cells was stimulated by direct co-culture with KG-1 cells. In contrast, indirect co-culture had little effect on BM cell proliferation (Figure 1A,B). KG-1 cell proliferation was not affected by the co-culture with BM cells (Figure 1A,B). MSCs are known to undergo accelerated differentiation during in vitro culture [2,3,4]. There were no major differences in the morphology between the cells co-cultured with and without KG-1 cells (Figure 1C). Therefore, we used flow cytometry to examine the expression of PDGFR-α and Sca-1 as well as of MSC-specific markers in mice [29,30]. The proportion of cells expressing PDGFR-α and Sca-1 was significantly higher in the cells co-cultured with KG-1 cells (BM cells + KG-1 cells) than in those cultured without KG-1 cells (BM cells) (Figure 1D). We further examined the effect of co-culture on multi-lineage differentiation ability. Chondrogenic differentiation was induced after co-culture, and the expression of Sox9 was observed in BM cells (Figure 1E). These data suggest that co-culturing BM cells with KG-1 cells promotes their proliferation and chondrogenic differentiation abilities.

### 2.2. Direct Mesenchymal-Hematopoietic Cell Interaction Promotes the Stemness of MSCs

**Figure 2:**
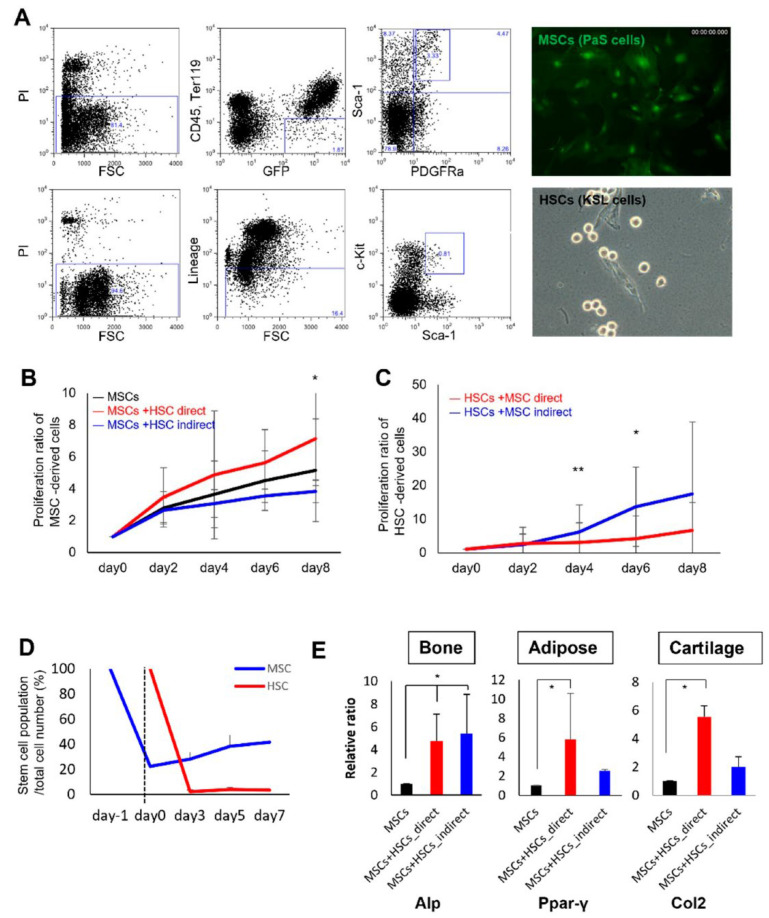
Clonal assay of MSCs after co-culture with HSCs. (**A**) Representative flow cytometric profiles of MSCs (upper, CD31^−^, CD45^−^, Ter119^−^, Sca-1^+^_,_ and PDGFR-α^+^ cells) and HSCs (bottom, c-Kit^+^, Lin^−^, and Sca-1^+^ cells). Microscopic views of MSCs (upper) and HSCs (bottom). (**B**,**C**) Cell proliferation of MSCs (**B**) and HSCs (**C**) 2, 4, 6, and 8 days after co-culture (*n* = 6). * *p* < 0.05 and ** *p* < 0.01 between direct and indirect co-culture. (**D**) MSC and HSC population in whole mesenchymal and hematopoietic cells. MSCs were sorted on day −1, and we waited until they attached to the bottom of the dish on day 0. After co-culture of MSCs and HSCs for 3, 5, and 7 days, cells were collected, sorted based on EGFP expression, and both cell types were detected using stem cell markers by flow cytometry. (**E**) Real-time PCR analysis of mesenchymal lineage marker expression in MSCs following a 3-week treatment with mesenchymal differentiation media after co-culture with HSCs (*n* = 5). * *p* < 0.05 vs. MSCs (-HSCs).

To re-confirm the effect of hematopoietic–mesenchymal cell interaction on MSCs, we evaluated changes in the MSC properties during co-culture with HSCs. The MSCs from mice expressing EGFP and HSCs from C57BL/6 mice were purified by flow cytometry (Figure 2A). The proliferation of MSCs was promoted by direct co-culture with HSCs, while indirect co-culture failed to affect proliferation (Figure 2B). Interestingly, hematopoietic cells showed a marked increase in cell number, which was especially observed during indirect co-culture (Figure 2C). To examine their proportions in the mesenchymal and hematopoietic cells proliferated by co-culture, the MSCs and HSCs were analyzed by flow cytometry using the MSC markers PDGFR-α/Sca-1 and the HSC markers Lin/Sca-1/c-Kit. MSC marker-positive cells showed a slight tendency to increase with time from day 0, while the population of HSCs greatly decreased immediately after co-culture (Figure 2D). Furthermore, the MSCs were evaluated for their multipotency after co-culture. After the induction of osteogenic, adipogenic, and chondrogenic differentiation, the expression of each differentiation marker was upregulated (Figure 2E). These data suggest the presence of a signal when transitioning from HSCs to MSCs.

### 2.3. Co-Culture with HSCs Promotes Collagen Synthesis and Adipogenic Differentiation

**Figure 3:**
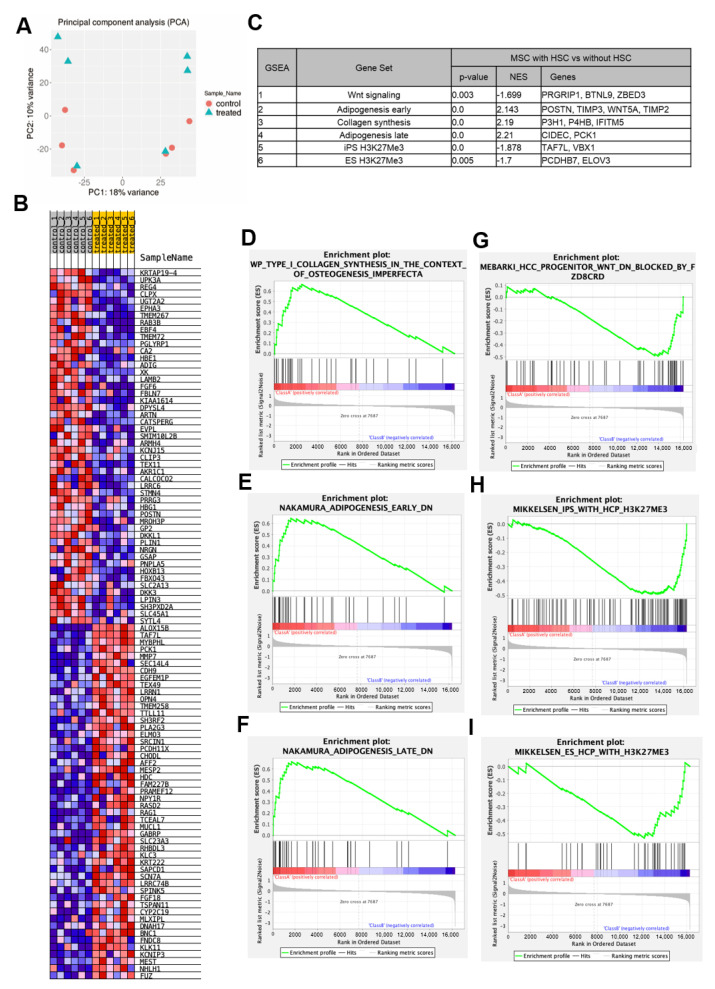
Gene expression analysis comparing MSCs cultured alone with those co-cultured with HSCs. (**A**) Principal component analysis (PCA) analysis of 12 samples. Orange circles: control MSC, blue triangles: MSC co-cultured with HSC. (**B**) Heatmap of differentially expressed genes between the MSC and HSC co-culture group (treated) and MSC−only group (control). (**C**) List of pathways/genes that were highly expressed in the MSC and HSC co-culture group. (**D**–**I**) Gene Set Enrichment Analysis (GSEA) of microarray.

To examine the effects of HSC signaling on the MSCs in more detail, we examined the gene expression profile of MSCs after co-culture with HSCs using microarray analysis. In the MSC and HSC co-culture group (treated) and MSC-only group (control), the upregulation of transcription factors was observed (Figure 3A,B). These MSCs expressed SRCIN1, BNC1, and PCK1. SRCIN1 regulates cell expansion and migration [31,32]. BNC1 is involved in the positive regulation of oocyte maturation and may also play a role in the early development of embryos [33,34]. PCK1 regulates adipogenesis via its protein kinase activity [35,36] (Figure 3C). Considering these results, HSCs may have the potential to maintain the undifferentiation properties and metabolism in MSCs. We also analyzed differentially expressed genes (DEGs) using TCC [37] and bayseq [38]. However, no DEGs were observed between the MSC-only and MSC and HSC co-culture groups. Gene set enrichment analysis (GSEA [39,40]) was also performed to detect the signal trends because no significant differences in gene expression were observed under single-factor comparison. In particular, gene expression rarely differed between the C5_ontology gene sets and C7_immunologic signature gene sets. As a result of analysis with the C2_curated gene sets, which are hallmark gene sets, a significant difference was found in the genes enriched in the following terms: ‘Type I collagen synthesis’ (Figure 3D), ‘Adipogenesis’ (Figure 3E,F), ‘Wnt blocked by Frizzled’ (Figure 3G), and ‘H3K27me3’ (Figure 3H,I), which were inhibited in the co-cultured MSCs. The GSEA analysis results supported that HSC signals might affect MSC properties.

### 2.4. MSCs Lacking Mpl Tend to Have Enhanced Differentiation Activity

**Figure 4:**
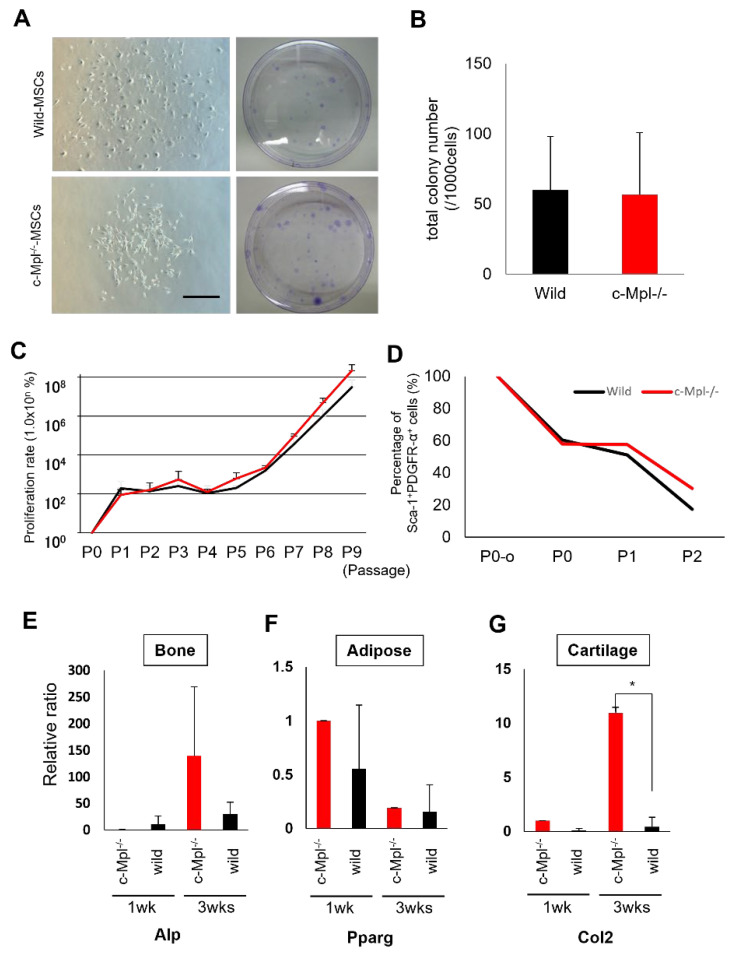
c-Mpl-deficient MSCs have normal proliferation and differentiation potential. (**A**) Phase-contrast images of cultures of c-Mpl-WT and c-Mpl-KO MSCs (left panels) and cells after 14 days of culture in CFU-F assay (right panels, 100 mm dishes). (**B**) Total numbers of CFU-Fs counted on day 14 (*n* = 15). Scale bar = 1 mm. (**C**) Growth curves for wild-type and c-Mpl^−/−^ mouse-derived MSCs (*n* = 3 per group). (**D**) Percentages of PDGFR-α- and Sca-1-positive cells in the cultured cells. (**E**–**G**) Real-time PCR analysis of tri-lineage marker expression (mAlp, (**E**); mPparg, (**F**); mCol2, (**G**)) in MSCs following 1- and 3-week treatments with mesenchymal differentiation media. * *p* < 0.05 c-Mpl^−/−^ MSCs vs. wild-type MSCs (*n* = 5).

MPL signaling is reportedly one of the direct mechanisms that maintain the undifferentiated state of HSCs. To determine the effect of the stemness signal from c-Mpl-deficient mice on MSCs, we analyzed cells from c-Mpl knockout mice. First, MSCs were harvested from c-Mpl-deficient and wild-type mice to examine the effects of HSC deficiency on MSCs. The c-Mpl-deficient mouse-derived MSCs showed a comparable colony-forming ability to the cells from the wild-type mice in the CFU-F assay (Figure 4A,B). When the harvested cells were subcultured, there was no significant difference in proliferative capacity or in the proportion of PDGFR-α/Sca-1-positive cells between the two cell types (Figure 4C,D). Furthermore, the multipotency of the MSCs was examined by real-time RT-PCR for marker differentiation in the chondrocytes, osteoblasts, and adipocytes after the induction of differentiation. Surprisingly, a significant increase in chondrogenic marker expression was observed in the c-Mpl-deficient mouse-derived MSCs. Although there was no significant difference in the expression of osteogenic and adipogenic markers between the two groups, the levels of these markers tended to be higher in the MSCs from the c-Mpl-deficient mice (Figure 4E–G). These results suggest that the c-Mpl-deficient MSCs exhibit enhanced differentiation activities in vitro.

### 2.5. c-Mpl Gene Deficiency Affects the Maintenance of Ectopic Bone

**Figure 5:**
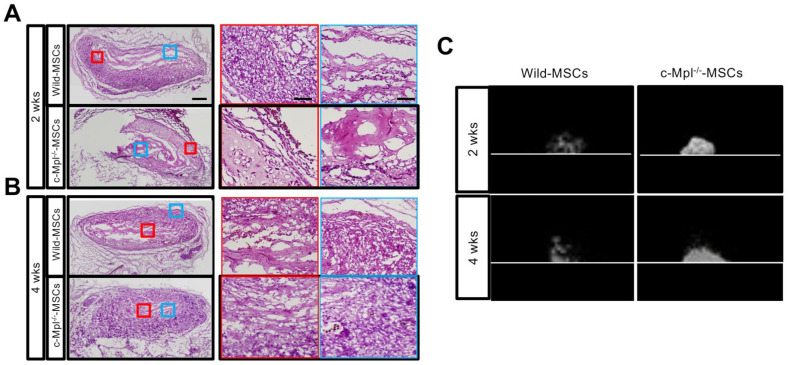
c-Mpl deficiency correlated with the differentiation ability of MSCs. (**A**,**B**) Hematoxylin and eosin staining shows ectopic bone formation at 2 weeks (**A**) and 4 weeks (**B**) after transplantation (left panel: low magnification; right panel: high magnification) (*n* = 3). Representative data are presented. (**C**) µCT findings of ectopic bone at 2 and 4 weeks after transplantation. At two weeks after transplantation, a high-intensity signal was detected in the group with c-Mpl-deficient MSCs (*n* = 3). Representative data are presented.

The osteogenic properties of MSCs from c-Mpl-deficient and wild-type mice were evaluated in vivo. MSCs were seeded in a collagen sponge containing BMP-2, cultured overnight, and implanted subcutaneously on the back of mice. c-Mpl-WT and c-deficient MSCs showed bone formation early after transplantation (Figure 5A,B). However, the c-Mpl-deficient mouse-derived MSCs promoted bone formation earlier than the wild-type MSCs did (Figure 5C). Hematoxylin and eosin staining showed the presence of grafts at both 2 and 4 weeks.

### 2.6. The c-Mpl in HSCs Regulates MSC Stem Cell Properties

**Figure 6:**
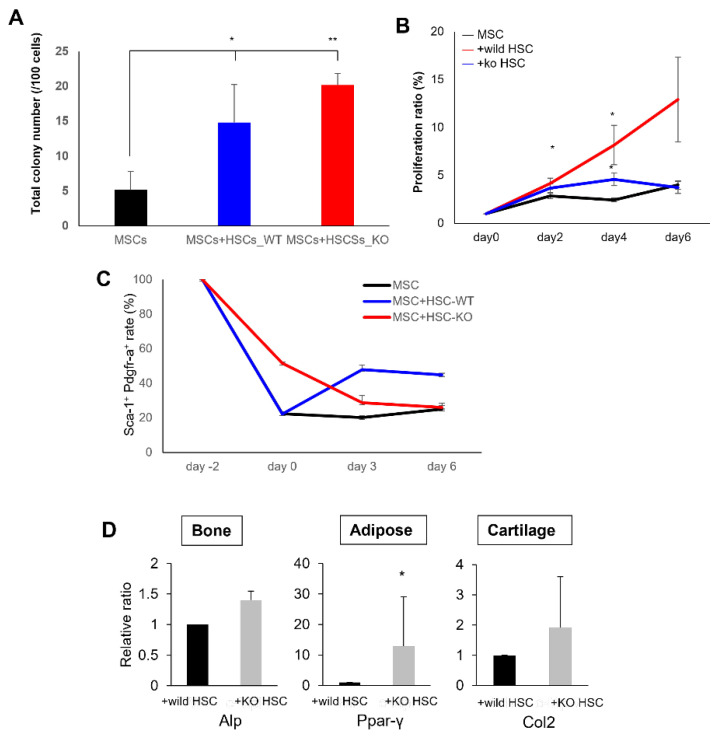
The c-Mpl in HSCs regulates the properties of MSCs**.** (**A**) Total numbers of CFU-Fs counted on day 14 after co-culture with HSCs (c-Mpl-WT and c-Mpl-KO). (**B**) Proliferation rate of MSCs after co-culture with HSCs (c-Mpl-WT and c-Mpl-KO) for 2, 4, and 6 days. * *p* < 0.05 (*n* = 5). (**C**) Percentages of PDGFR-α- and Sca-1-positive cells in the MSCs co-cultured with HSCs (c-Mpl-WT and c-Mpl-KO). (**D**) Real-time PCR analysis for tri-lineage marker expression in MSCs following a 2-week treatment with mesenchymal differentiation media after co-culture with c-Mpl-WT or c-Mpl-KO HSCs. * *p* < 0.05, ** *p* < 0.01 (*n* = 5).

To examine the relationship between the stemness of the HSCs and the stemness signal from the HSCs to the MSCs, wild-type MSCs were co-cultured with HSCs from wild-type mice or c-Mpl-deficient mice deficient in HSCs [41]. To demonstrate that the properties of MSCs are affected by c-Mpl signal deficiencies, wild-type MSCs were co-cultured with wild-type or c-Mpl-deficient HSCs. The colony-forming ability of MSCs was not affected by c-Mpl deficiency after co-culture with HSCs (c-Mpl-wild type (WT) and c-Mpl-knock out (KO)) (Figure 6A). However, the co-culture with the HSCs from c-Mpl-deficient mice failed to stimulate MSC proliferation (Figure 6B). In the MSCs that did not co-culture, although the Sca-1- and PDGFR-α positive rates decreased immediately after culture, they remained almost unchanged from day 0 to day 6 (Figure 6C). In the wild-type MSCs co-cultured with wild-type HSCs, although the positive rates of Sca-1 and PDGFR-α temporarily decreased from day −2 to day 0, they recovered on day 3 and were maintained at around 40% (Figure 6C). The multipotency of MSCs co-cultured with HSCs from c-Mpl-deficient or wild-type mice was examined by real-time RT-PCR. Adipogenic marker levels were significantly higher in MSCs co-cultured with HSCs from c-Mpl-deficient mice. The levels of chondrocyte differentiation markers tended to be higher in MSCs co-cultured with HSCs from c-Mpl-deficient mice, although the difference was not statistically significant. The expression of osteogenic markers was similar between the two groups (Figure 6D). Therefore, we suggest that in HSCs, c-Mpl affects the maintenance of the proliferative ability in MSCs. 

## 3. Discussion

In this study, we showed that the hematopoietic cell line KG-1 stimulated the proliferation of MSCs via cell-to-cell contact. In addition, HSCs not only stimulated the proliferation of MSCs, but also promoted their tri-lineage differentiation. These results clearly indicate the existence of stemness signals that are directly transduced from hematopoietic cells to mesenchymal cells. 

HSC stemness itself plays an important role in these stemness signals because the disruption of stemness in HSCs via the deletion of c-Mpl affected the proliferation and differentiation of MSCs in co-culture assays. MSCs co-cultured with c-Mpl-deficient HSCs showed less proliferative activity as well as increased tri-lineage differentiation activities, contrary to our expectation. These results can be interpreted in two ways. First, c-Mpl-deficient HSCs provide a niche-like environment in which MSCs are kept in a relatively dormant state and can maintain their multipotency (hypothesis #1). Second, MSCs tend to differentiate and lose their proliferative activity when co-cultured with c-Mpl-deficient HSCs (hypothesis #2). The transplantation of c-Mpl-deficient MSCs results in earlier bone formation and degradation at a later time point. This result indicates that hypothesis #2 is more plausible. We also studied the effects of Tpo–c-Mpl signaling inhibition in MSCs. Although c-Mpl deficiency has been shown to affect hematopoietic function, no reference has been made to MSCs [42,43,44,45,46]. Our data provide evidence for comparable proliferation, a similar proportion of clonogenic cells, and similar reductions in the proportion of PDGFR-α/Sca-1 cells between c-Mpl^−/−^ MSC clones and wild-type ones. In contrast, tri-lineage differentiation ability tended to be higher in the c-Mpl^−/−^ MSCs. This can be attributed to the effect of the c-Mpl^−/−^ HSCs co-existing with MSCs in mice before collection and considering the effect of the c-Mpl^−/−^ HSCs on the MSCs (Figure 3). However, the possibility that cell-autonomous mechanisms underlie the enhanced differentiation activity cannot be completely excluded at this point. 

To dissect the effects of c-Mpl deficiency in HSCs and MSCs on the stemness of MSCs, cells from both c-Mpl-deficient and WT mice were co-cultured, and the proportion of PDGFR-α/Sca-1 cells was examined at several time points. While the MSCs from the c-Mpl-deficient and WT mice showed similar results, the HSCs from both mice exhibited different patterns. The number of WT HSCs increased and maintained the proportion of PDGFR-α/Sca-1 cells in the MSCs after a decrease on day 0. However, the MSCs that were co-cultured with the c-Mpl-deficient HSCs showed a gradual decrease in the proportion of PDGFR-α/Sca-1 cells. These results suggest that c-Mpl deficiency in HSCs, but not in MSCs, has a great impact on the maintenance of stemness in MSCs when they are co-cultured with HSCs. 

To determine whether MSC properties are affected by HSC signals, microarray analysis was performed on the changes in the gene expression of the MSCs after they were co-cultured with HSCs. Although it was shown that the MSC properties were affected by co-culture with HSCs, the expression of each gene showed no statistically significant difference between the MSCs cultured with, or without, HSCs. We also analyzed DEGs, but no DEGs were observed between the MSCs cultured with, or without, HSCs. GSEA analysis was also performed to detect the signal trend because there were no significant differences in gene expression found during the single-factor comparison. Analysis with C2_curated gene sets, the hallmark gene sets in the Molecular Signatures Database (MsigDB) [39,40,47], revealed a significant difference in adipogenesis and Wnt signaling. We consider the following as the reasons for the observation of differences in only a limited number of pathways: First, gene expression shows higher batch-to-batch variation in bulk arrays in general. Second, heterogeneity may still exist, even within separated cells; even if a gene is highly expressed in a certain cell population, it does not necessarily mean that all MSCs express the gene at a high level [48]. Therefore, it is possible that the microarray data of co-cultured MSCs do not completely correspond to the GSEA data. These are possible limitations to the interpretation of the microarray data analysis.

KG-1 is a myeloblast cell line derived from human acute leukemia. Taking into consideration the genetic characteristics of KG-1, it is not possible to certify the mesenchymal-hematopoietic interaction accurately. However, some papers have reported that myeloblastic cell lines such as the KG-1 mimic some but not all aspects defined in primary CD34+ cell populations [49]. On the other hand, HeLa, which is a cell line derived from human cervical cancer, is used not only in cancer research but also in many different fields of research, such as immunological research and infectious diseases. Therefore, we thought that KG-1 could be used for this study. In addition, we think that they mimic our hypothetical signal communication with MSC-HSC, which is the essence of hematopoietic-mesenchymal signals, because myeloblasts are differentiated from hematopoietic progenitor cells.

Taken together, this study verified the stemness signals from hematopoietic cells to mesenchymal cells. In addition, this indicates that the stemness of HSCs is important for maintaining the stemness of MSCs. Our findings may help to establish a culture method to expand MSCs with high stemness by taking advantage of stemness signals, thereby contributing to the development of regenerative medicine.

## 4. Materials and Methods

### 4.1. Mice

C57BL/6JJcl mice (6–12 weeks old) were purchased from CLEA Japan Inc. (Tokyo, Japan). C57BL/6-Tg(CAG-EGFP)10sb/J mice, transgenic mice that ubiquitously express EGFP under the control of the CAG promoter, and C57BL6/J-Mpl<hlb219/J> mice, chemically induced mutation mice with the loss of the c-Mpl expression via *ENU* (N-ethyl-N-nitrosourea) and that were 6–12 weeks of age, were purchased from Charles River Laboratory (Yokohama, Japan). The mice were maintained under specific pathogen-free conditions in our animal facility at the University of Tokyo. All the animal experiments were conducted in accordance with the guidelines of the University of Tokyo. The care and use of the animals in this study followed the guidelines of the University of Tokyo and the laws and notifications of the Japanese government. All the animal experiments were approved by the Animal Care and Use Committee of the University of Tokyo (ethics permission #622, animal experiment permission # P09-026).

### 4.2. Preparation of BM Cell Suspension

The femurs, tibias, and ilium were dissected and crushed with scissors and a pestle. The crushed bones were gently washed once in HBSS+ (Hanks-balanced salt solution supplemented with 2% FBS, 10 mM HEPES, and 100 U/mL penicillin/0.1 mg/mL streptomycin solution), and the solution was filtered through a cell strainer (BD Falcon, #2350, Glendale, AZ, USA) and discarded. Bone fragments were collected and incubated for 1 h at 37 °C in 20 mL of DMEM (Thermo Fisher Scientific, Waltham, MA, USA) containing 0.2% collagenase (Fujifilm-Wako, Tokyo, Japan), 10 mM HEPES, and 100 U/mL penicillin/0.1 mg/mL streptomycin solution. The suspension was filtered with a cell strainer (BD Falcon, #2350) to remove debris and bone fragments and collected by centrifugation at 400× *g* for 5 min at 4 °C. The red blood cells were lysed in the suspension with the addition of 1 mL of ice-cold sterile H_2_O (Sigma-Aldrich, Tokyo, Japan) for 6 s. Immediately after the 6-s lysis, 1 mL of 2× PBS (diluted product from Sigma-Aldrich) with 4% (vol/vol) FBS was added to quench the reaction. Red cell lysis buffer (ACK Lysing Buffer, Grand Island, NY, USA) may be used as an alternative. The suspension was filtered through a 70 µm cell strainer (Falcon, BD). These serial procedures were described in previous reports [29,30].

### 4.3. Antibody Staining and Flow Cytometry

The cells were suspended in ice-cold HBSS+ at 1–5 × 10^7^ cells/mL and then stained for 30 min on ice with the following monoclonal antibodies: biotinylated or APC-conjugated PDGFR-α (Cat# 17-1401-81) and c-Kit, PE-conjugated Sca-1 (Ly6A/E. Cat# 12-5981-82), PE-Cy7-conjugated lineage markers (Lin: CD3e (Cat# 25-0031-82), CD11b (Cat# 25-0112-82), B220 (Cat# 25-0460-82), Gr-1 (Cat# 25-5931-82), CD45 (Cat# 25-0451-82)), CD31 (Cat# 25-0311-82), and TER-119 (Cat# 25-5921-82). All monoclonal antibodies were purchased from eBioscience (San Diego, CA, USA). Flow cytometry analysis and sorting were performed using a FACS AriaIII Fusion (BD Biosciences, San Jose, CA, USA). Propidium iodide (PI) fluorescence was measured, and a live cell gate was defined to exclude cells positive for PI. Additional gates for MSCs were defined as positivity for PDGFR-α and Sca-1 and negativity for CD31, CD45, and TER119; HSCs were defined as positivity for c-Kit and Sca-1 and negativity for Lin, according to the fluorescence intensity of the isotype control.

### 4.4. Cell Culture

Traditional MSC adherent culture was performed as previously described [50]. Sorted MSCs were seeded on a plastic tissue culture dish with IMDM containing 20 ng/mL FGF-2 (Kaken Pharmaceutical Co., Ltd., Tokyo, Japan), 20% FBS, and 100 U/mL penicillin/0.1 mg/mL streptomycin solution at 37 °C with 5% CO_2_ and maintained with fresh medium being added every 3–4 days for 1–2 weeks.

### 4.5. CFU-F Assay

Approximately 1 × 10^3^ sorted cells were seeded on a 100 mm dish with IMDM containing 20% FBS and 100 U/mL penicillin/0.1 mg/mL streptomycin solution at 37 °C with 5% CO_2_. Adherent cell clusters containing > 40 cells were counted as colonies after 10 days of culture.

### 4.6. Differentiation Cultures

To induce adipocyte differentiation, sub-confluent cells were cultured with three cycles of adipogenic induction medium/adipogenic maintenance medium, supplemented with the adipogenic induction/adipogenic maintenance SingleQuot kit medium (Lonza, Ltd., Basel, Switzerland). Each cycle consisted of feeding the sub-confluent cells with the induction medium for 3 days, followed by 3 days of culture in the maintenance medium. After 14 days, the cells were harvested using TRI Reagent (Sigma-Aldrich Co., LLC, Waltham, MA, USA). For chondrogenic differentiation, 2 × 10^4^–2.5 × 10^5^ cells were seeded into a 15 mL conical tube. The tube was centrifuged at 400× *g* for 5 min at 25 degrees, and the supernatant was aspirated. The cells were resuspended in 1 mL differentiation basal chondrogenic medium, supplemented with Chondrogenic SingleQuot kit medium (Lonza, Ltd.), and centrifuged at 400× *g* for 5 min, and the medium was aspirated. The cells were resuspended in 1 mL differentiation basal chondrogenic medium supplemented with the Chondrogenic SingleQuots kit medium, TGF-β3 (10 ng/mL; Lonza, Ltd.), and BMP-6 (500 ng/mL; R&D Systems, Inc., Minneapolis, MN, USA), and centrifuged at 150× *g* for 5 min at room temperature. The pellet was maintained in differentiation basal medium that was changed every 3–4 days for 2 weeks. After 3 weeks, cell clumps were harvested using TRI Reagent. To induce osteoblast differentiation, sub-confluent cells were cultured in differentiation basal osteogenic medium supplemented with osteogenic SingleQuots kit medium (Lonza, Inc.) for 14 days. The cells were then harvested using TRI Reagent. 

### 4.7. Co-Culture of MSCs with Hematopoietic Cells

For mesenchymal cells, whole BM cells and Sca-1^+^ and Pdgfr-α^+^ cells (MSCs) derived from mice labeled with green fluorescent protein were used for differentiation from hematopoietic cells. For hematopoietic cells, Lin^−^, Sca-1^+^, and c-Kit^+^ cells (HSCs) derived from wild-type or c-Mpl-deficient mice and the human acute myeloid leukemia cell line (KG-1), which are poorly differentiated and close to HSCs, were used. For co-culture, 6.0 × 10^4^ BM cells and 1.4 × 10^5^ KG-1 cells were cultured in 100 mm dishes, or 3.0 × 10^3^ MSCs and 7.0 × 10^3^ HSCs were cultured in 12-well dishes with IMDM containing 20% FBS. For direct co-culture, MSCs were seeded and cultured on the plate at 37 °C for 24 h, and then HSCs or KG-1 cells were added. For indirect co-culture, MSCs were seeded on the bottom of Transwells and cultured at 37 °C for 24 h, and then HSCs or KG-1 cells were seeded on the inserts. The co-culture periods were 2, 4, 6, and 8 days, and both cell types were harvested at each time point. The harvested cells were counted using fluorescence microscopy (Leica Microsystems, Wetzlar, Germany). On day 6 of co-culture, MSCs were induced for tri-lineage differentiation.

### 4.8. RNA Isolation and RT-PCR

RNA was isolated from 1.0–5.0 × 10^4^ cells, converted to cDNA, and amplified (PrimeScript 1st strand cDNA synthesis kit and RT-PCR kit, TaKaRa Bio Inc., Kusatsu, Japan). Reverse transcription was performed by incubation at 65 °C for 5 min followed by immediate cooling on ice. The PCR amplification conditions for the resulting cDNA were 35 cycles of 94 °C for 30 s, 58 °C for 45 s, and 68 °C for 45 s, in which the 68 °C step was increased by 5 s every cycle after 10 cycles. The reaction products were resolved by electrophoresis on 2% agarose gel and visualized with ethidium bromide.

### 4.9. Real-Time RT-PCR Analysis for Selected mRNAs

Approximately 200 ng of total RNA was used to synthesize double-stranded cDNA by reverse transcription (Super Script III; Invitrogen, Waltham, MA, USA) according to the manufacturer’s instructions. cDNA was analyzed by real-time PCR using Universal SYBR Green PCR Master Mix (Invitrogen). The reaction conditions were 50 °C for 2 min, 95 °C for 10 min, and then 40 cycles of 95 °C for 15 s followed by 60 °C for 1 min. For the relative quantitation of gene expression, mouse-specific GAPDH and Hprt1 were used as internal controls. All of the other PCR primer sequences are listed in Table 1.

### 4.10. Microarray

When the MSCs were co-cultured with HSCs for 6 days, total RNA was extracted with TRI reagent following the manufacturer’s instructions (Sigma-Aldrich Co., LLC, Waltham, MA, USA). RNA quality was determined by measuring the absorbance at 260 nm, and the RNA integrity was checked by agarose gel electrophoresis. Briefly, 10 µg of total RNA was used to generate first-strand cDNA. After second-strand synthesis, biotinylated and amplified RNA was purified with RNeasy (Qiagen, Hilden, Germany) and quantitated by spectrophotometry. The GeneChip Mouse Genome 430 2.0 Array (Afymetrix Inc., Santa Clara, CA, USA) was used in this study. This array contains probe sets for 45,000 transcripts and clones. After hybridization, the microarray plate was washed, scanned, and analyzed using the GeneChip Operating Software (GCOS, v1.1.1, Affymetrix Japan K.K, Tokyo, Japan). Data are available in the GEO repository profile of the NCBI database (GSE186250, https://www.ncbi.nlm.nih.gov/geo/query/acc.cgi?acc=GSE186250, accessed on 22 May 2022). These data were imported into Microsoft Excel for downstream analysis [51].

### 4.11. GSEA of Microarray

GSEA software developed by the UC San Diego and Broad Institute (Cambridge, MA, USA) was used. The curated gene dataset (C2) in MSigDB v7.4 was used to extract enriched terms from gene expression data [40].

### 4.12. In Vivo Bone and Stroma Assay

For the analysis of in vivo bone and stroma formation, MSCs were harvested from the bone marrow of wild-type and c-Mpl-deficient mice, and CD45^−^/CD31^−^/Ter119^−^/Sca-1^+^/PDGFR-α^+^ cells were obtained using FACS AriaIII Fusion. Cells from three different donors were used. One hundred thousand cells were cultured overnight in a 3% cell matrix sponge containing 2 μg/mL BMP-2 and implanted subcutaneously into 6-week-old male C57BL/6 mice. The implants were removed after 2 and 4 weeks, fixed, subjected to CT scanning, decalcified, and paraffin-embedded (*n* = 3). Sections were stained with hematoxylin and eosin and analyzed for bone formation as previously described [52]. 

### 4.13. Statistical Analysis

The data are presented as the mean ± standard error of the mean (SEM), and each independent experiment was reproduced three to five times. The comparison between two conditions was performed using an unpaired *t*-test. One-way repeated-measures ANOVA were applied to identify significant differences among the conditions or groups. When a significant difference was observed, the data were subjected to a post hoc analysis. *p* < 0.05 was considered significant.

## 5. Conclusions

In this study, the stemness signal from such hematopoietic cells to the mesenchymal cells was verified. In addition, it was indicated that the stemness of HSCs is important to maintain the stemness of MSCs. If a culture method to expand MSCs with high stemness can be established by taking advantage of stemness signals in the future, then it is expected to be used to make dramatic developments in regenerative medicine.

## Figures and Tables

**Table 1:** All other PCR primer sequences are listed.

Gapdh	FW	5′- CCACTAACATCAAATGGGGTGAGG -3′
	RV	5′- TACTTGGCAGGTTTCTCCAGGC -3′
Hprt	FW	5′- TCAGTCAACGGGGGACATAAA -3′
	RV	5′- GGGGCTGTACTGCTTAACCAG -3′
Col2a1	FW	5′- TTGAGACAGCACGACGTGGAG -3′
	RV	5′- AGCCAFFTTGCCATCGCCATA -3′
Sox9	FW	5′- TCTCCTAATGCTATCTTCAAGGCG -3′
	RV	5′- TGCTCAGTTCACCGATGTCCAC -3′
Alp	FW	5′- CACAATATCAAGGATATCGACGTGA -3′
	RV	5′- ACATCAGTTCTGTTCTTCGGGTACA -3′
Bglap	FW	5′- GGGCAATAAGGTAGTGAACAG -3′
	RV	5′- GCAFCACAFFTCCTAAATAGT -3′
Adipoq	FW	5′- TGTTCCTCTTAATCCTGCCCA -3′
	RV	5′- CCAACCTGCACAAGTTCCCTT -3′
Pparg	FW	5′- ACCACTCGCATTCCTTTGAC -3′
	RV	5′- TGGGTCAGCTCTTGTGAATG -3′

## Data Availability

The microarray supporting the findings of this study has been deposited in GEO under accession code GSE186250. Data are further available for download and interactive browsing in their processed form at https://www.ncbi.nlm.nih.gov/geo/query/acc.cgi?acc=GSE186250 (accessed on 22 May 2022).
